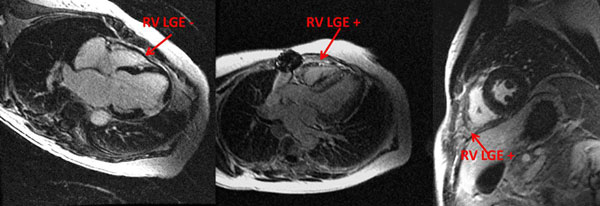# Sarcoidosis; is it confined to just the LV? An RV LGE study

**DOI:** 10.1186/1532-429X-16-S1-P280

**Published:** 2014-01-16

**Authors:** Huma Y Samar, Diane V Thompson, Mark Doyle, Ronald B Williams, June A Yamrozik, Sahadev T Reddy, Moneal Shah, Robert W Biederman

**Affiliations:** 1 Cardiac MRI, Allegheny General Hospital, Pittsburgh, Pennsylvania, USA

## Background

Sarcoidosis is a multisystem disease. Involvement of the left ventricle (LV) in Sarcoidosis detected by CMR in the form of early (EGE) and late gadolinium enhancement (LGE) is well validated. Autopsy studies have shown abnormalities in the right ventricle (RV) as well, albeit less frequently than the LV. There have also been several case reports of sarcoidosis mimicking ARVD suggesting predominant RV involvement in those cases Objective: We sought to determine if there was evidence of RV involvement in patients with Sarcoidosis that could be detected via early and late gadolinium enhancement on CMR

## Methods

A retrospective review was performed on a database of 122 patients (mean age 62, 62 males, 55 females) referred for evaluation of cardiac sarcoidosis between 2002 and 2013 at a single center. All scans were performed using a 1.5T scanner (GE, Milwaukee, Wisconsin). Via that database, we found 37 patients demonstrated phenotypic cardiac sarcoidosis in the form of early and late gadolinium enhancement in the LV myocardium that was corroborated by the clinical presentation. The CMR images of these 37 patients (mean age 52; 24 male and 13 female) were then reviewed by a single experienced operator to assess for the presence of EGE or LGE in the RV by standard visual analysis, using the SA, 3- and 4-chamber views obtained at 2 and 10 minutes post contrast administration (0.5 mmol/kg, Multihance, Bracco, Princeton, NJ). In this study T2 was not uniformly performed. LVEDV, LVESV and LVEF measurements were obtained using semi-automated analysis software(Medis Qmass 7.4.26.0) and were available in 31 patients (81%). Ventricular dimensions were available in all 37 patients. Patient characteristics were compared between the groups that demonstrated RV involvement with those that did not. Presence of LGE in the RV was also correlated with volumetric data where available and ventricular dimensions. Categorical data was presented as counts and percentages and analyzed using the chi-square test. The independent samples t-test or Mann-Whitney test was used to assess the difference between patients with respect to continuous variables. Data was analyzed using PASW Statistics, version 18.0 (IBM SPSS Inc.).

## Results

Out of the 37 patients that showed the presence of cardiac sarcoidosis in the LV, RV enhancement was seen in 18 (49%). There were no significant difference in LV or RV metrics between patients that exhibited RV enhancement versus those that did not. RV enhancement did not correlate with differences in LV volumes, LVEF, LV or RV dimensions.

## Conclusions

Our proof of concept study demonstrates that RV involvement does occur in a significant number of patients along with LV involvement in cardiac sarcoidosis and this can be detected via early and late gadolinium enhancement supporting a less myopic view of sarcoid. As to whether RV involvement portends worse outcome is ongoing work.

## Funding

Internal.

**Figure 1:**